# Medical Clinical Institute, Genoa, Italy

**Published:** 1885-04

**Authors:** A. Lagorio

**Affiliations:** Chiavari, Italy


FIBRINOUS PNEUMONIA.

Among the many interesting cases in the Medical Clinical Institute at Genoa, directed by Prof. Maragliano, there were two patients affected with fibrinous pneumonia, which, for the course and result of the disease and also for the method of treatment employed, deserve special notice. The history of one of them is as follows:

A man, 44 years old, a blacksmith by trade, suffered some twenty years ago from a right-sided pneumonia. Recovery was complete. He never abused alcoholic drinks. On the 12th of January, last, he was taken with a rigor, pain in the right mammary region and the epigastrium, accompanied by a violent cough, with which he expectorated sputa, tinged with blood. He complained of intense cephalalgia. On the 14th, he entered the *clinique;* his temperature in the axilla was 39.6° C. On examination, he was found to be a man of robust constitution, in the left lateral decubitus. The right half of the thorax dilated less than the left, the number of respirations was 50 per minute. On percussion, nothing abnormal was noted anteriorly: posteriorly, there was found hypophonesis from the angle of the scapula to the base of the right thorax, hypophonesis at the left base, increased vocal fremitus, bronchial breathing and crepitant râles.

Heart, spleen and liver, normal; nothing abnormal in the urine, except an abundance of uroerythrin. The patient was submitted, as is usual in this *clinique*, to the stimulant treatment,—that is, a tablespoonful of a potion, containing fifteen per cent. of alcohol, every hour, and 25 centigrams of caffeine every four hours. On the 15th, the fourth day of the disease, the temperature was 40° C., pulse 112, respiration 48. No change in the respiratory organs. On the 16th (fifth day of the disease) the hypophonesis of the right base posteriorly changed into dullness— the same numerous crepitant râles persisting at the left base, pulse 90, respiration 48. On the 17th (sixth day of the disease), the temperature, taken hourly, varied from 38.5° to 39.5° C. On palpation, bronchial fremitus was observed anteriorly, on the right and left sides. Dry cups were applied. 18th, the patient passed the night in delirium. The temperature varied from 37.7° to 39.6° C. On the right side posteriorly and in the lower region, there were heard crepitant and subcrepitant râles, and small mucous râles. To the left, subcrepitant râles—the bronchial fremitus persisted anteriorly. Dry cups were applied, and a hypodermic injection of hydrochlorate of quinine, as a heart tonic, was exhibited. At noon the pulse was 120 and small. Hypodermic injection of sulphuric ether was given. 19th (eighth day of disease), delirium. The temperature varied from 38.6° to 39.6° C.; pulse, 116. The bronchial fremitus persisted anteriorly. Posteriorly to the right, there were heard coarse mucous râles, and coarse subcrepitant râles at the left. Injection of ether. At noon the pulse 114, the patient was brighter, answered questions, the temperature was 38° C. An enema of three grains of

*antipyrine* was given, and after four hours the temperature fell to 37.8°, continuing at this four hours. The vocal fremitus persisted and dry cups were applied. 20th (ninth), temperature 38.2°. The bronchial fremitus has disappeared. Evening, temperature 39°, pulse 112, small and irregular. The bronchial fremitus reappeared. A grain of ether was injected, dry cups were applied, and three grains of antipyrine were given in an enema. The temperature fell after six hours to 37.4° C., and continued at this seven hours.

21st (tenth), the same symptoms of the respiratory organs were persistent. Inhalations of oxygen were given. 22d (eleventh), the bronchial fremitus had disappeared. There was an abundant expectoration of muco-purulent sputa, nummular and puriform. A grain and a half of bisulphate of quinine was prescribed, and a tablespoonful of Marsala wine hourly. 23d (twelfth), temperature 37.6°. Symptoms unchanged. There were prescribed every three hours inhalations of three cylinders of medicated compressed air passed through a saturated solution of iodoform in the essential oil of turpentine, also a tablespoonful hourly of three grains of oil of turpentine in 300 grains of gum arabic emulsion. 24th (thirteenth), nothing new. 25th (fourteenth), pericardiac fremitus and a friction sound was noticed. 26th (fifteenth), diminished respiration without pus. A slight improvement, the temperature did not exceed 38.8° C. Secretion of urine increased (2,400 c.c. in 24 hours). 27th (16th), temperature 38.9°, pulse 120, respiration 48. Harsh breathing anteriorly at the right and left. Posteriorly at the right the dull sound was changed into tympanitic, the mucous râles were less numerous than on the preceding days. The patient was given a mixed diet of meat, eggs and milk. Continued to take inhalations of medicated compressed air, and took four pills of strychnine, of a milligrain each, daily.

28th, 29th and 30th (17th, 18th and 19th day of disease), nothing new.

31st (20th), the improvement continued; expectorated freely; the temperature did not exceed 38.2° C. in the evening.

February 1st (21st), pericardial symptoms disappeared; the improvement continued.

2d, 3d and 4th (22d, 23d and 24th), nothing new.

5th (25th), râles fast disappearing. Temperature did not exceed 38° in the evening. 6th (26th), the oil of turpentine internally was stopped, and four tablespoonfuls of cod liver oil with half per cent. of pure creosote were given.

7th to 15th (27th to 35th), the patient was apyretic, the appetite is fair, the cough less frequent, the expectoration muco-salivary, with some muco-purulent sputa. Posteriorly to the left the diminution of resonance at the base continued, with harsh respiration and some small mucous râles. The resonance had returned to the right, the râles were few.

The history of the other patient was about the same.

On these two patients, Prof. Maragliano gave a clinical lecture. He examined the different conditions capable of continuing the fever in a fibrinous pneumonia. The fever may be continuous, sub-continuous and intermittent. The conditions maintaining it are, the formations of cirrhotic parts, when the fever has fallen by crisis; from the eighth to the ninth day, there lights up again the gray hepatization, or the purulent infiltration, or the pulmonary abscess, the gangrene, or the caseous change of the exudates. But the *pulmonary abscess* gives rise to a remittent fever, a purulent expectoration and all the symptoms of the formation of a cavity in the parenchyma of the lungs. The *gangrene.* Besides the typical remittent fever with sweats and rigors, the gangrene is characterized by a breath of cadaveric odor, and by a characteristic expectoration, while the *caseous degeneration* maintains the fever of an intermittent type; in this the râles are subcrepitant, and the organic wasting progresses rapidly. In the gray hepatization of purulent infiltration, we have a sub-continuous fever, typhoid state, general wasting, and small and large râles in the inspiratory as well as the expiratory acts.

The diagnosis made in this patient was bilateral fibrinous pneumonia, localized at the base of the inferior lobes, passing into the stage of purulent infiltration. Congestion and œdema of the non-inflamed pulmonary part—dry pericarditis.

This result and course of the fibrinous pneumonia in these two patients is undoubtedly due to their organic constitutions. In one of them, besides old age (65 years), existed a marked vascular atheroma, due to chronic alcoholism, while in the other can be admitted a less resistance of his organism to some special conditions not explanable.

In these cases, it is to be noted how, by the application of dry cups, we can avoid imminent dangers of pulmonary œdema, and how beneficial are the inhalations of medicated compressed air, which not only excite the bronchial mucous membranes to expel the secretions accumulated in the lungs; but while they disinfect the respiratory surface, they prevent the absorption of infecting materials, which besides the local harm, cause without a doubt a great general injury. In these cases the inhalations caused a modification of the expectoration, but they lowered the temperature, and in one of the two patients there is great hope of a complete restoration of the pulmonary parenchyma.

A. LAGORIO.

Chiavari, Italy.

